# Effects of ****ω****-3 Polyunsaturated Fatty Acids on Plasma Proteome in Rett Syndrome

**DOI:** 10.1155/2013/723269

**Published:** 2013-12-09

**Authors:** Claudio De Felice, Alessio Cortelazzo, Cinzia Signorini, Roberto Guerranti, Silvia Leoncini, Alessandra Pecorelli, Thierry Durand, Jean-Marie Galano, Camille Oger, Gloria Zollo, Barbara Montomoli, Claudia Landi, Luca Bini, Giuseppe Valacchi, Lucia Ciccoli, Joussef Hayek

**Affiliations:** ^1^Neonatal Intensive Care Unit, University Hospital Azienda Ospedaliera Universitaria Senese (AOUS), Viale M. Bracci 16, 53100 Siena, Italy; ^2^Child Neuropsychiatry Unit, University Hospital AOUS, Viale M. Bracci 16, 53100 Siena, Italy; ^3^Department of Medical Biotechnologies, University of Siena, Via A. Moro 2, 53100 Siena, Italy; ^4^Department of Molecular and Developmental Medicine, University of Siena, Via A. Moro 2, 53100 Siena, Italy; ^5^Institut des Biomolécules Max Mousseron (IBMM), UMR 5247, CNRS/UM1/UM2/ENSCM, BP 14491, 34093 Montpellier Cedex 5, France; ^6^Department of Life Science, University of Siena, Via A. Moro 2, 53100 Siena, Italy; ^7^Department of Life Sciences and Biotechnology, University of Ferrara, Via Borsari 46, 44100 Ferrara, Italy; ^8^Department of Food and Nutrition, Kyung Hee University, 1 Hoegi-dong, Dongdaemun-gu, Seoul 130-701, Republic of Korea

## Abstract

The mechanism of action of omega-3 polyunsaturated fatty acids (**ω**-3 PUFAs) is only partially known. Prior reports suggest a partial rescue of clinical symptoms and oxidative stress (OS) alterations following **ω**-3 PUFAs supplementation in patients with Rett syndrome (RTT), a devastating neurodevelopmental disorder with transient autistic features, affecting almost exclusively females and mainly caused by sporadic mutations in the gene encoding the methyl CpG binding protein 2 (MeCP2) protein. Here, we tested the hypothesis that **ω**-3 PUFAs may modify the plasma proteome profile in typical RTT patients with *MECP2* mutations and classic phenotype. A total of 24 RTT girls at different clinical stages were supplemented with **ω**-3 PUFAs as fish oil for 12 months and compared to matched healthy controls. The expression of 16 proteins, mainly related to acute phase response (APR), was changed at the baseline in the untreated patients. Following **ω**-3 PUFAs supplementation, the detected APR was partially rescued, with the expression of 10 out of 16 (62%) proteins being normalized. **ω**-3 PUFAs have a major impact on the modulation of the APR in RTT, thus providing new insights into the role of inflammation in autistic disorders and paving the way for novel therapeutic strategies.

## 1. Introduction

Omega-3 polyunsaturated fatty acids (*ω*-3 PUFAs) have received an increasing attention by the scientific community and the society as well due to their status of natural molecules with a number of claimed positive effects on a large variety of conditions, in particular in the prevention of cardiovascular disease. Most common *ω*-3 PUFAs are eicosapentaenoic acid (EPA) and docosahexaenoic acid (DHA) found in fish oil and *α*-linolenic acid C18:3 n-3, derived from plants. The hypotriglyceridemic effect is the best defined metabolic action of *ω*-3 PUFAs [1], with a mechanism likely to be related to activation of peroxisome proliferator-activated receptors [2]. A host of other potential beneficial effects of *ω*-3 PUFAs has been suggested. Besides the demonstrated or claimed beneficial cardiovascular effects (reduction of susceptibility to ventricular arrhythmia [3]; antithrombogenic and antioxidant effect [4]; retardation of the atherosclerotic plaque growth by reduced expression of adhesion molecules and platelet-derived growth factor [4]; promotion of endothelial relaxation by induction of nitric-oxide production; and mild hypotensive effect [5]), a more general anti-inflammatory effect, either direct or indirect has been reported [6–8]. However, the molecular mechanisms underlying the *ω*-3 PUFAs effects in the regulation of the inflammatory process are still poorly understood and an emerging major field of research [9–11]. Several factors may indeed contribute to limit our understanding in this specific area of the lipid metabolism, including the type of PUFAs, formulation, dose, duration, age and underlying condition of the target subjects, and—last but not least—lack of objective measurement of effects. However, a relatively recent report in adult smoker subjects [12] demonstrates that *ω*-3 PUFAs are able to affect plasma protein expression by regulating acute phase response (APR).

Rett syndrome (RTT) is a devastating neurodevelopmental disorder mainly caused by sporadic mutations in the gene encoding the methyl-CpG binding protein 2 (MeCP2) protein. RTT affects almost exclusively females with an average frequency of 1 : 10,000 female live births and is considered to be the second commonest cause of severe cognitive impairment in this gender [13]. RTT has been formerly included in the autism spectrum disorders (ASDs), although evident differences with autism exist [14] and is no longer classified in the ASDs group. Nevertheless, transient autistic features are always present in the RTT natural history. Therefore, this relatively rare disease actually offers a good opportunity to test the effects of *ω*-3 PUFAs in an objective way in an autistic condition, given that (1) a persistent redox imbalance has been demonstrated [15]; (2) *ω*-3 PUFAs have been suggested to reduce phenotypical severity and improve the redox balance in supplemented patients at several stages of their clinical history [15]; (3) an impaired cholesterol metabolism has been very recently demonstrated in a *Mecp2*-null mouse model of RTT, with statin treatment leading to improvement of motor symptoms and conferring increased longevity [16]; and (4) an unexplained hypercholesterolemia has been reported by our group in RTT patients [17].

Since only one study has examined the plasma proteome of RTT [18], here we hypothesized that *ω*-3 PUFAs may have an impact on the RTT plasma proteome by modulating the APR.

## 2. Materials and Methods

### 2.1. Subjects

The study included a total of 24 female patients with a clinical diagnosis of typical RTT (mean age: 14.4 ± 8.0 years, range 4–33 years) with demonstrated *MECP2* gene mutations [i.e., T158 M (*n* = 5), C-terminal deletions (*n* = 4), R255X (*n* = 4), R270X (*n* = 3), R133C (*n* = 2), early truncating mutations (*n* = 1), large deletions (*n* = 1) other mutations (*n* = 4)]. Clinical stages distribution was stage I (*n* = 4), stage II (*n* = 6), stage III (*n* = 7), and stage IV (*n* = 7). RTT diagnosis and inclusion/exclusion criteria were based on the recently revised RTT nomenclature consensus [19, 20]. All the patients were admitted to the Rett Syndrome National Reference Centre of the University Hospital of the Azienda Ospedaliera Universitaria Senese (Head: Professor J. Hayek). Blood samplings in the patients' group were performed during the routine follow-up study at hospital admission, while the samples from the control group were carried out during routine health checks, sports, or blood donations, obtained during the periodic clinical checks. The healthy control subjects (*n* = 24) were gender (given that over 98% of RTT patients are females, we selected a female control group) and age matched (mean age: 14.4 ± 8.2 years, range 4.1–33 years). All the examined subjects were on a typical Mediterranean diet. The study was conducted with the approval by the Institutional Review Board and all informed consents were obtained from either the parents or the legal tutors of the enrolled patients.

### 2.2. Study Design

The aim of the present study was to assess the effects on the plasma proteome of a supplementation already tested to be effective in the clinical setting [21–23]. Therefore, as a consequence we did not include a placebo arm for ethical reasons and limited the study to three subjects population, that is, healthy controls, “untreated” Rett syndrome, and *ω*-3 PUFAs supplemented Rett syndrome.

### 2.3. *ω*-3 PUFAs Supplementation

Administered *ω*-3 PUFAs were in the form of fish oil (Norwegian Fish Oil AS, Trondheim, Norway, product number HO320-6; Italian importer: Transforma AS Italia, Forlì, Italy; Italian Ministry registration code: 10 43863-Y) at a dose corresponding to DHA, 71.9 ± 13.9 mg/kg b.w./day and EPA, 115.5 ± 22.4 mg/kg b.w./day, with a total *ω*-3 PUFAs of 242.4 ± 47.1 mg/kg b.w./day. Use of EPA plus DHA in RTT was approved by the AOUS Ethical Committee.

The dose used in this specific RTT girls cohort is likely to be 5 to 6 times higher than the standard one, which is typically 2 g per day in adult subjects. The rationale for it is contained in a prior paper [21], in which we proposed a very high dose in Rett syndrome. After several attempts, the final dose per kg/day was found empirically in the clinical setting.

### 2.4. Sample Collection

All samplings from RTT patients and healthy controls were carried out around 8 AM after overnight fasting. Blood was collected in heparinized tubes and all manipulations were carried out within 2 h after sample collection.

### 2.5. Sample Preparation

The blood samples were centrifuged at 2400 g for 15 min at 4°C; the platelet poor plasma was saved; and the buffy coat was removed by aspiration. Plasma samples were stored at −70°C until assay.

### 2.6. Two-Dimensional (2D) Gel Electrophoresis

2-DE was performed according to Görg et al. [24], and samples containing 60 *μ*g of protein as determined by Bradford [25] were denatured with a solution containing 10% of sodium dodecyl sulfate (SDS) and 2.3% of dithiothreitol (DTT) heated to 95°C for 5 min. The sample was then combined solubilizing buffer composed by 8 M urea, 2% of 3-[(3-cholamidopropyl)-dimethylammonio]-1-propanesulfonate (CHAPS), 0.3% DTT, and 2% of immobilized pH gradient (IPG) buffer and loaded into 18 cm IPG strips 3–10 non linear on an Ettan IPGphor (GE Healthcare) apparatus system and rehydrated for 7 h. Isoelectric focusing (IEF) was carried out for a total of 32 kV h. The strips were first equilibrated with a buffer containing 50 mM Tris-HCl, pH 8.8, 6 M urea, 2% w/v SDS, 30% v/v glycerol, and 1% w/v DTT for 15 min; then they were equilibrated again with the same buffer described above, except it contained 4% w/v iodoacetamide instead of DTT. The second dimension was performed on an Ettan Daltsix Electrophoresis system (GE Healthcare). IPG strips were embedded at the top of a 1.5 mm thick vertical polyacrylamide gradient gel (8–16% T) using 0.5% w/v agarose and run at a constant current of 40 mA/gel at 20°C. Each sample was carried out in triplicate under the same conditions.

### 2.7. Image Analysis

Images of gels were analyzed using ImageMaster 2D Platinum v7.0 software (GE Healthcare). The reference gel for each group (i.e., healthy controls, untreated RTT, and RTT after *ω*-3 supplementation) was defined and used for the comparative analyses. The background was subtracted from all gels using the average-on-boundary method. Spot volume was expressed as a ratio of the total protein percentage volume (%V) detected from the entire gel to minimize differences between gels (gel normalization), for pooling them. Only spots appearing in all gels of the same group were matched with those of the reference gel.

### 2.8. Proteins Identification

After mass spectrometry compatible silver staining [26], a spot-picking list was generated and exported to Ettan Spot Picker (GE Healthcare). The spots were excised and delivered into 96-well microplates where they were destained and dehydrated with acetonitrile (ACN) for subsequent rehydration with trypsin solution. Tryptic digestion was carried out overnight at 37°C. Each protein spot digest (0.75 mL) was spotted into the MALDI instrument target and allowed to dry. Then, 0.75 mL of the instrument matrix solution (saturated solution of *α*-cyano-4-hydroxycinnamic acid in 50% ACN and 0.5% v/v trifluoroacetic acid) was applied to dried samples and dried again. Mass spectra were obtained, as described [27], using an ultrafleXtreme MALDI-ToF/ToF (Bruker Corporation, Billerica, MA, USA). After tryptic peptide mass acquisition, mass fingerprint searching was carried out in Swiss-Prot/TREMBL and NCBInr databases using MASCOT (Matrix Science, London, UK, http://www.matrixscience.com/).

### 2.9. Data Analysis

All variables were tested for normal distribution (D'Agostino-Pearson test) and data were presented as median and interquartile range unless otherwise. Statistical analysis for protein expressed differently in the groups was carried out using Student's *t*-test and one-way ANOVA test. Bonferroni-corrected significance levels were used for multiple *t*-tests. Unmatched spots or spots with significantly different %V were considered “differently expressed” in the groups. Comparisons between differently expressed proteins of untreated RTT and RTT after *ω*-3 supplementation were evaluated using either Mann-Whitney rank sum test or Kruskal-Wallis test. The effects of small population sizes on possible type I (*α*)/type II (*β*) errors in the data interpretation were examined using a sampling size algorithm. A two-sided *P* < 0.05 was considered to indicate statistical significance, The MedCalc version 12.1.4 statistical software package (MedCalc Software, Mariakerke, Belgium) was used.

## 3. Results

The expression of 16 proteins, mainly related to the APR, was found to be changed at the baseline level in the untreated RTT patients.

**Figure 1:**
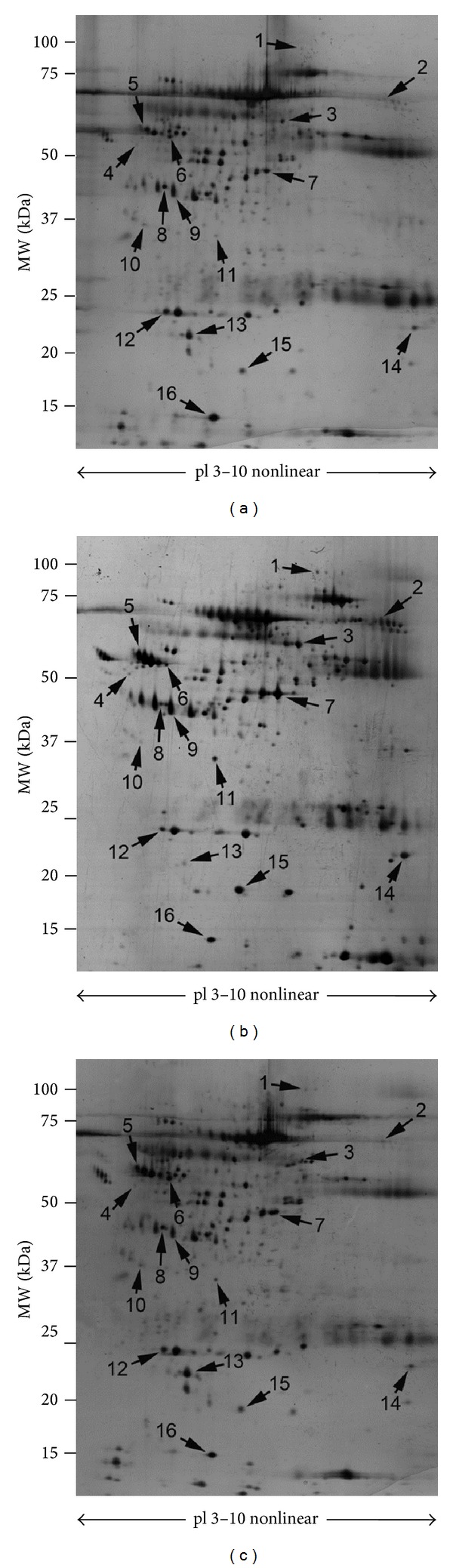
Silver-stained 2-DE gel of proteins from healthy control (a), untreated RTT (b), and RTT after *ω*-3 PUFAs supplementation (c). 60 *μ*g of total protein was subjected to nonlinear IPG strips, with a pH range from 3 to 10, followed by SDS-polyacrylamide gradient gel (8–16% T) electrophoresis. Molecular mass and pI markers are indicated along the gels. Numbers denote the mass spectrometry-identified protein spots which are listed in Tables 1 and 2. The same protein spots are reported in 3 representative gels from (a) healthy controls, (b) untreated RTT patients; and (c) *ω*-3 PUFAs supplemented RTT patients.

**Figure 2:**
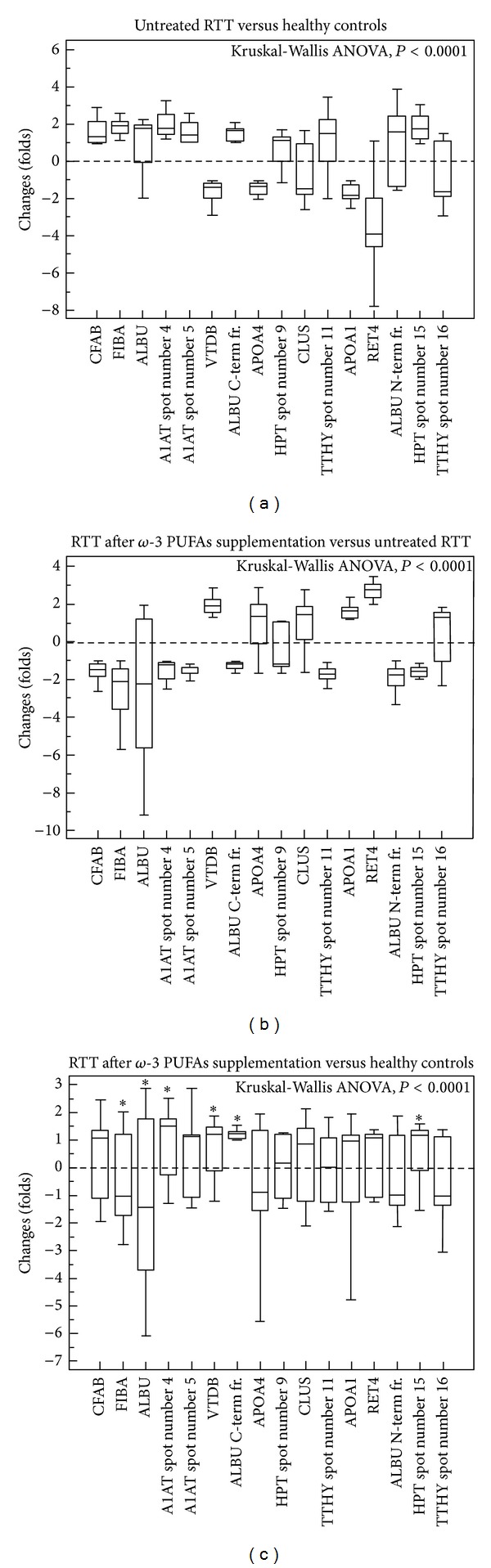
Plasma proteins expression as a function of *ω*-3 PUFAs supplementation in *MECP2*-mutated girls with classical RTT. (a) RTT patients before supplementation: expression levels are compared to healthy controls; (b) RTT patients after supplementation: expression levels are compared to untreated RTT; (c) RTT patients after supplementation: expression levels are compared to healthy controls. Data are expressed as box-and-whiskers plots. Box-and-whisker plots representation was used in a quite unconventional fashion. Our aim was to try to visually display relative changes in the expression of individual protein as compared to healthy controls. The variables represented correspond to relative changes: a value of 0 represents no changes in expression as compared to controls; positive values indicate protein overexpression, while negative values indicate protein underexpression. Significance of the changes were detected at ImageMaster analysis; therefore, this figure corresponds to a graphical device in order to visually detect the observed protein changes. Results of Kruskal-Wallis ANOVA are indicated. Asterisks in panel (c) indicates persistently overexpressed (top symbols) and persistently underexpressed proteins following *ω*-3 PUFAs supplementation as compared to control levels of expression.

As compared to healthy controls, the whole RTT group showed significant increase in 10 protein spots [i.e., complement factor B (CFAB), fibrinogen alpha chain (FIBA), serum albumin (ALBU, spots number 3, number 7 C terminal fragment and number 14 N terminal fragment) alpha-1-antitrypsin (A1AT, spots number 4 and number 5), haptoglobin (HPT, spots number 9 and number 15), and transthyretin (TTHY spot number 11)] and decrease in 6 protein spots [i.e., vitamin D-binding protein (VTDB), apolipoprotein A4 (APOA4), clusterin (CLUS), apolipoprotein A1 (APOA1), retinol-binding protein 4 (RET4), and transthyretin (TTHY spot number 16)] (Table 1 and Figures 1(a), 1(b), and 2(a)). A full list of the known biological functions for the identified plasma proteins is shown in Table 2.

Following *ω*-3 PUFAs supplementation, the expression of 10 out of 16 (62%) proteins was found to be comparable to that of control subjects (Figure 2(c)). In particular, after *ω*-3 PUFAs, plasma proteins expression levels were comparable to those of the control population, with the exception of persistent overexpression of A1AT (spot number 4), VTDB (spot number 6), ALBU C Terminal fragment (spot number 7), and HPT (spot number 15) and the persistent underexpression of FIBA (spot number 2) and ALBU (spot number 3).

By comparing the plasma protein profile of treated RTT with the one before treatment, significant decreases in 9 protein spots previously overexpressed before treatment, including CFAB, FIBA, ALBU (spots number 3 and number 14), A1AT, HPT, and TTHY (spot number 11) were reported, whereas significant increases were observed for 5 protein spots that were underexpressed before the *ω*-3 PUFAs supplementation and included VTDB, APOA4, CLUS, APOA1, and RET4. After *ω*-3 PUFAs supplementation, the levels ALBU (spot number 7) and TTHY (spot number 16) remained unchanged compared to untreated RTT (Table 1 and Figures 1(a), 1(c), and 2(b)).

## 4. Discussion

The mechanism of action of *ω*-3 PUFAs is a major area of research, which has led in the last two decades to the discovery of protectins, resolvins and maresins, and all lipid mediators involved in the active resolution of the inflammatory process [28].

Our results indicate that *ω*-3 PUFAs are able to modulate plasma protein expression in RTT, having a major impact on the modulation of the APR, with a partial (approximately 62%) rescue of the protein changes observed at baseline. These findings well fit with the known anti-inflammatory properties of this fatty acids family [29].

The APR is a highly conserved adaptive mechanism [30] and is a core part of the innate immune response. Profound changes occur in the plasma proteome as the consequence of APR, reflecting a highly regulated process as part of a more generalized reprogramming of signaling events under the influence of cytokines. The involved proteins (i.e., APR proteins) are known to be predominantly synthesized in the liver, and the signaling events result in either an upregulation or downregulation of APR proteins. More than 200 plasma proteins are known to vary in the APR, some of which may control tissue damage and participate in tissue repair, although their role still remains speculative [31].

Omega-3 PUFAs have multiple health benefits mediated at least in part by their anti-inflammatory actions. A recent paper [29] demonstrated that EPA and DHA are competitors for arachidonic acid (AA) in binding to the 5-lipooxygenase enzyme, since *ω*-3 PUFAs displace AA in membrane phospholipids, reducing the production of AA-derived eicosanoids (i.e., prostaglandin E_2_) while increasing those generated from *ω*-3 PUFAs activation. As reported in several trials, *ω*-3 PUFAs supplementation is able to reduce plasma and urine levels of eicosanoids such as leukotriene E_4_ [32–35]. Besides the anti-inflammatory effects based on the interruption of the AA cascade, *ω*-3 PUFAs impart their anti-inflammatory effects via a decreased activation of nuclear factor-kappa B (NF-KB), a potent inducer of the production of proinflammatory cytokines, including interleukin-6 and tumor necrosis factor-*α*. Overall, enrichment of cellular membranes with *ω*-3 PUFAs disrupts dimerization and recruitment of toll-like receptor-4, which might contribute to anti-inflammatory effects by downregulation of NF-KB activation [29]. Further evidence demonstrates that *ω*-3 PUFAs can repress lipogenesis and increase generation of resolvins and protectins, ultimately leading to reduced inflammation. Finally, the effects of EPA and DHA include their ability to increase secretion of adiponectin, an anti-inflammatory adipokine [36]. When considering the effects of *ω*-3 PUFAs on the cellular function, their direct modulation of G-protein-coupled receptor is noteworthy and might contribute to the anti-inflammatory properties. At the same time, *ω*-3 PUFAs show a direct regulation on gene expression vianuclear receptors and transcription factors, which are in turn modulated by cytoplasmic lipid binding proteins transporting these fatty acids into the nucleus. Regulation of *ω*-3 PUFAs on gene expression could explain the altered protein expression that we reported on the RTT plasma proteome and is in line with previous findings in adult smokers following a short course (i.e., 5 weeks) of *ω*-3 PUFAs enriched diet [12]. In this latter study, proteins related to the antioxidant, anti-inflammatory, and antiatherosclerotic properties of HDL were upregulated, in contrast with down-regulation of complement activation and APR proteins.

In particular, in our study major changes for proteins involved in APR (CFAB, FIBA, ALBU (spot number 3 C terminal fragment and spot number 14 N terminal fragment), A1AT (spots number 4 and number 5) and HPT (spots number 9 and number 15)), immunity, unfolded protein response (CLUS), blood coagulation (FIBA), transport pathways (TTHY (spots number 11 and number 16), RET4 and VTDB), and lipid metabolism (APOA4 and APOA 1) were evidenced at the proteome analysis of plasma samples from a population of patients with typical RTT at different stages and harboring a variety of *MECP2* gene mutations. Overexpressed plasma proteins in the unsupplemented RTT patients were mainly related to APR and underexpressed spots corresponded to negative APR proteins, unfolded protein response, and proteins involved in the lipid metabolism. Our data indicated that *ω*-3 PUFAs almost completely rescued the APR detected at the baseline.

The molecular mechanisms of the *ω*-3 PUFAs action remain only partially understood and include changes in membrane structures and gene expression, direct interactions with ion channels, and alterations in eicosanoid biosynthesis [28]. EPA and DHA, key *ω*-3 PUFAs, have been reported to compete with AA for the conversion by cytochrome P450 enzymes, thus resulting in the formation of alternative, physiologically active, metabolites, given that cytochrome P450 enzymes are known to efficiently convert EPA and DHA to epoxy and hydroxy metabolites (17,18-epoxyeicosatetraenoic, and 19,20-epoxydocosapentaenoic acid, resp.) [37], which could likely mediate some of their beneficial effects [38].

The present study strongly suggests that the main beneficial action of *ω*-3 PUFAs (or their secondary metabolites) in RTT is the modulation of an unrecognized subclinical inflammatory status and fits well with the known anti-inflammatory properties of this fatty acids family and suggesting that the super-family of the multiple actions attributed to *ω*-3 PUFAs could be attributable to the APR modulation.

## 5. Conclusion

A subclinical inflammatory state has been previously reported in autistic subjects with significant changes in inflammation-related proteins [39]. Overall, our findings of a subclinical APR in RTT that can be modulated by a dietary supplementation of *ω*-3 PUFAs provide new insights into the role of inflammation in autistic disorders and support the role of *ω*-3 PUFAs as key nutraceuticals [40].

## Figures and Tables

**Table 1:** Identification results of proteins differentially expressed in basal and in RTT before and after *ω*-3 PUFAs supplementation.

Experimental								Data bank exploration					
Spot	Protein name	Untreated RTT			RTT after *ω*-3 supplementation			UniProt ID	Entry name	Mascot score	Sequence coverage (%)	Peptides matches	Theoretical pI/Mr (kDa)
		ANOVA (*P* < 0.05)	Fold change*	Protein expression	ANOVA (*P* < 0.05)	Fold change**	Protein expression						
1	Complement Factor B	0.042	+1.58	Upregulated	0.030	−1.40	Downregulated	P00751	CFAB	102	21	11/24	6.67/86.4
2	Fibrinogen alpha chain	0.009	+2.07	Upregulated	0.008	−2.04	Downregulated	P02671	FIBA	120	30	25/86	5.70/95.6
3	Albumin	0.016	+1.67	Upregulated	0.012	−1.66	Downregulated	P02768	ALBU	242	38	22/33	5.92/71.3
4	Alpha-1-antitrypsin	0.001	+1.97	Upregulated	0.019	−1.33	Downregulated	P01009	A1AT	235	60	26/69	5.37/46.8
5	Alpha-1-antitrypsin	0.003	+1.73	Upregulated	0.005	−1.47	Downregulated	P01009	A1AT	106	25	10/18	5.37/46.8
6	Vitamin D-binding protein	0.002	−1.52	Downregulated	0.001	+1.85	Upregulated	P02774	VTDB	123	35	14/40	5.40/54.5
7	Albumin (C terminal fragment)	0.027	+1.46	Upregulated	—	—	—	P02768	ALBU	79	18	9/25	5.92/71.3
8	Apolipoprotein A-IV	0.038	−1.45	Downregulated	0.029	+1.46	Upregulated	P06727	APOA4	107	36	13/48	5.28/45.3
9	Haptoglobin	0.010	+1.17	Upregulated	0.027	−1.15	Downregulated	P00738	HPT	58	16	9/23	6.13/45.8
10	Clusterin	0.013	−1.34	Downregulated	0.009	+1.50	Upregulated	P10909	CLUS	131	24	8/8	5.89/53.0
11	Transthyretin	0.012	+1.91	Upregulated	0.015	−1.79	Downregulated	P02766	TTHY	113	59	6/10	5.52/15.9
12	Apolipoprotein A-I	0.043	−1.50	Downregulated	0.024	+1.52	Upregulated	P02647	APOA1	253	70	27/69	5.56/30.7
13	Retinol-binding protein 4	0.001	−2.56	Downregulated	0.001	+2.59	Upregulated	P02753	RET4	112	46	8/18	5.76/23.3
14	Albumin (N terminal fragment)	0.021	+1.73	Upregulated	0.014	−1.78	Downregulated	P02768	ALBU	143	24	14/23	5.92/71.3
15	Haptoglobin	0.001	+1.81	Upregulated	0.003	−1.55	Downregulated	P00738	HPT	72	21	10/31	6.13/45.8
16	Transthyretin	0.044	−1.32	Downregulated	—	—	—	P02766	TTHY	120	68	7/15	5.52/15.9

Experimental section includes Spot ID, protein name, and the significant threshold level (*P* < 0.05). Variation in spot intensities represented by fold is expressed as ratio of RTT to control and as a ratio of RTT after *ω*-3 supplementation to RTT. Positive fold changes denote proteins upregulated, while negative fold changes denote decrease of protein expression levels. — denotes the absence of significant changes. Data bank exploration of proteins includes SwissProt data bank accession numbers and entry name, the Mascot score, the sequence coverage, the number of peptides matching the protein sequence, and theoretical pI/Mr × 10^−3^. *Comparisons versus healthy controls; **Comparisons versus untreated RTT.

**Table 2:** Biological functions for the identified proteins.

Proteins overexpressed in untreated RTT	
Protein	Biological function
Complement factor B	Immune system and complement system regulation
Fibrinogen alpha chain	Coagulation and signal transduction
Serum albumin	Transport, regulation of colloidal osmotic pressure, and platelet activation
Alpha-1-antitrypsin (spot number 4)	Acute phase response, coagulation, and proteases inhibition
Alpha-1-antitrypsin (spot number 5)	Acute phase response, coagulation, and proteases inhibition
Serum albumin (C terminal fragment)	Transport, regulation of colloidal osmotic pressure, and platelet activation
Haptoglobin (spot number 9)	Acute phase response and hemoglobin binding
Transthyretin (spot number 11)	Thyroid hormone binding and transport
Serum albumin (N terminal fragment)	Transport, regulation of colloidal osmotic pressure, and platelet activation
Haptoglobin (spot number 15)	Acute phase response and hemoglobin binding

Proteins underexpressed in untreated RTT	
Protein	Biological function

Vitamin D-binding protein	Vitamin D sterols carrier
Apolipoprotein A-IV	Lipid metabolism
Clusterin	Apoptosis, complement system regulation, and innate immunity
Apolipoprotein A-I	Lipid transport and metabolism
Retinol-binding protein 4	Retinol transport and metabolism
Transthyretin (spot number 16)	Thyroid hormone binding and transport

## Abbreviations

CFAB: Complement factor B
FIBA: Fibrinogen alpha chain
ALBU: Serum albumin
A1AT: Alpha-1-antitrypsin
VTDB: Vitamin D-binding protein
APOA4: Apolipoprotein A4
HPT: Haptoglobin
CLUS: Clusterin
TTHY: Transthyretin
APOA1: Apolipoprotein A1
RET4: Retinol-binding protein 4.
